# Statins Decrease Lung Inflammation in Mice by Upregulating Tetraspanin CD9 in Macrophages

**DOI:** 10.1371/journal.pone.0073706

**Published:** 2013-09-09

**Authors:** Yingji Jin, Isao Tachibana, Yoshito Takeda, Ping He, Sujin Kang, Mayumi Suzuki, Hanako Kuhara, Satoshi Tetsumoto, Kazuyuki Tsujino, Toshiyuki Minami, Takeo Iwasaki, Kaori Nakanishi, Satoshi Kohmo, Haruhiko Hirata, Ryo Takahashi, Koji Inoue, Izumi Nagatomo, Hiroshi Kida, Takashi Kijima, Mari Ito, Hideyuki Saya, Atsushi Kumanogoh

**Affiliations:** 1 Department of Respiratory Medicine, Allergy and Rheumatic Diseases, Osaka University Graduate School of Medicine, Suita, Osaka, Japan; 2 Department of Respiratory Medicine, the Second Affiliated Hospital, School of Medicine, Xi’an Jiaotong University, Xi’an, Shaanxi, China; 3 CREST, JST, Department of Immunopathology, WPI Immunology Frontier Research Center, Osaka University, Suita, Osaka, Japan; 4 Drug Research Division, Dainippon Sumitomo Pharma Co, Ltd, Osaka, Osaka, Japan; 5 Division of Gene Regulation, Institute for Advanced Medical Research, School of Medicine, Keio University, Shinjuku, Tokyo, Japan; University of Vermont, United States of America

## Abstract

Tetraspanins organize protein complexes in tetraspanin-enriched membrane microdomains that are distinct from lipid rafts. Our previous studies suggested that reduction in the levels of tetraspanins CD9 and CD81 may be involved in the progression of inflammatory lung diseases, especially COPD. To search for agents that increase the levels of these tetraspanins, we screened 1,165 drugs in clinical use and found that statins upregulate CD9 and CD81 in RAW264.7 macrophages. The lipophilic statins, fluvastatin and simvastatin, reversed LPS-induced downregulation of CD9 and CD81, simultaneously preventing TNF-α and matrix metalloproteinase-9 production and spreading of RAW264.7 cells. These statins exerted anti-inflammatory effects *in vitro* in wild-type macrophages but not in CD9 knockout macrophages, and decreased lung inflammation *in vivo* in wild-type mice but not in CD9 knockout mice, suggesting that their effects are dependent on CD9. Mechanistically, the statins promoted reverse transfer of the LPS-signaling mediator CD14 from lipid rafts into CD9-enriched microdomains, thereby preventing LPS receptor formation. Finally, upregulation of CD9/CD81 by statins was related to blockade of GTPase geranylgeranylation in the mevalonate pathway. Our data underscore the importance of the negative regulator CD9 in lung inflammation, and suggest that statins exert anti-inflammatory effects by upregulating tetraspanin CD9 in macrophages.

## Introduction

Pulmonary emphysema, a major manifestation of chronic obstructive pulmonary disease (COPD), is characterized by tissue destruction and airspace enlargement in the lung. As a result of exposure to cigarette smoke, which contains LPS, macrophages are persistently activated and infiltrate into the lung, producing inflammatory cytokines such as TNF-α and IL-6 and tissue-destructive proteases such as matrix metalloproteinase (MMP)-2, MMP-9, and MMP-12. In a major mechanism underlying COPD, cigarette smoke inactivates histone deacetylases (HDACs), resulting in sustained LPS-induced activation of macrophages [1]. Accumulating evidence has shown that COPD is frequently associated with age-related extrapulmonary comorbidities including cardiovascular diseases, type 2 diabetes, osteoporosis, and muscle atrophy [2,3]; consequently, COPD is projected to become the third commonest cause of death worldwide by 2020, but effective therapeutic agents have not been established. Importantly, persistent inflammation underlies the progression of COPD and related extrapulmonary disorders, suggesting that pulmonary emphysema and its comorbidities may have a common pathophysiologic mechanism [4]. Cells of the monocyte/macrophage lineage are likely to be key players because they can cause chronic inflammation in the arterial wall, adipose tissue, and bone, thereby contributing to the development of cardiovascular diseases, diabetes, and osteoporosis, respectively [5,6].

Proteins of the tetraspanin superfamily bind to its specific partners such as integrins, growth factor receptors, membrane proteases, and intracellular signaling molecules. By virtue of their characteristic structures, which span the membrane four times, tetraspanins can assemble dynamically to form membrane-bound multiprotein complexes in response to various stimuli [7,8]. In association with cholesterol and gangliosides, these complexes provide a lipid-rich platform designated tetraspanin-enriched microdomains (TEMs), which regulate signals essential for cell activation, adhesion, migration, and fusion, possibly by interacting with lipid rafts [9,10]. CD9 and CD81, two closely related tetraspanins, are abundantly expressed in monocytes/macrophages, suggesting that they play an important role in this cell lineage [11]. Previously we reported that mouse macrophages deficient in CD9 are strongly activated *in vitro* and cause enhanced lung inflammation *in vivo* when stimulated with LPS. In one proposed mechanism of action, CD9 negatively regulates LPS-induced macrophage activation by preventing CD14-dependent receptor assembly at the lipid raft [10]. Moreover, mice doubly deficient in CD9 and CD81 spontaneously develop pulmonary emphysema and osteoporosis, a phenotype akin to human COPD [12]. We and others reported that CD9 and CD81 in macrophages are downregulated by inflammatory stimuli including LPS, cigarette smoke extract, and the HDAC inhibitor trichostatin A (TSA) [10,12,13]. We also found that levels of these tetraspanins are decreased in blood monocytes from COPD patients (B Zhou and I Tachibana, unpublished data). These findings implicate downregulation of CD9 and CD81 in macrophage activation and resultant progression of COPD.

Anti-inflammatory agents that could prevent the cigarette smoke-induced activation of monocytes/macrophages would not only improve pulmonary dysfunction but also treat disorders comorbid with COPD. Therefore, upregulation of CD9 and CD81 could be a novel therapeutic approach. In this study, we screened more than 1,000 drugs that are currently in clinical use for their potential to upregulate CD9 and CD81 in macrophages. Among the drugs identified by the screen were statins, which inhibit the mevalonate pathway. We also propose novel anti-inflammatory mechanisms of statins that are dependent on CD9.

## Materials and Methods

### Ethics statement

Animal experiments were performed in accordance with the Osaka University guidelines on animal care. The protocol was approved by the Animal Experiments Committee of Osaka University. All procedures were under pentobarbital anesthesia, all efforts were made to minimize animal suffering, and mice were sacrificed using carbon dioxide (CO2).

### Mice

The generation of CD9 knockout (KO) mice [14] was described previously. The mice were backcrossed more than six generations into the C57BL/6J background and bred in a barrier facility. Nine- to 12-week-old CD9 KO mice and wild-type (WT) littermates matched for age and sex were randomized into groups in all experiments.

### Immunoblotting and immunoprecipitation

Cells were lysed in lysis buffer containing 1% NP-40, 20 mM Tris-HCl [pH 7.4], 150 mM NaCl, 2 mM EDTA, 2 mM PMSF, 10 µg/ml aprotinin, 10 µg/ml leupeptin, 1 mM orthovanadate and 50 mM NaF. Cell lysates were electrophoresed on SDS-PAGE and transferred to Immobilon-P membranes (Millipore). Membranes were probed with primary antibodies (Abs) followed by peroxidase-conjugated secondary Abs. Immunoreactive bands were visualized using Western Lightning^TM^ Chemiluminescent Reagent (PerkinElmer). The primary Abs were rat anti-mouse CD9 mAb (KMC8; BD Biosciences), hamster anti-mouse CD81 mAb (Eat2; UK-Serotec), rabbit anti-LAMP3 (CD63) polyclonal Ab (12632-1-AP; Proteintech), mouse anti-human CD9 mAb (MM2/57; Biosource), mouse anti-human CD81 mAb (JS64; Immunotech), rabbit anti-IκBα polyclonal Ab (9242; Cell Signaling Technology), rat anti-mouse integrin β2 subunit mAb (M18/2; BD Biosciences), rat anti-CD14 mAb (rmC5-3; BD Biosciences), rabbit anti-TLR4 polyclonal Ab (IMG577; Imgenex), mouse anti-flotillin-1 mAb (clone 18; BD Biosciences), and goat anti-CD45 polyclonal Ab (R&D Systems). For densitometry, blots were analyzed on a ChemiDoc^TM^ XRS Plus System using the Image Lab^TM^ Software (Bio-Rad). For immunoprecipitation, RAW264.7 cells were lysed in 0.5% NP-40 lysis buffer and cell lysates were incubated with anti-CD14 mAb or control IgG. Immune complexes were collected with protein G-Sepharose (Amersham Biosciences) and subjected to SDS-PAGE followed by immunoblotting using biotinylated anti-CD9 or anti-CD81 mAb and peroxidase-conjugated streptavidin (Zymed Laboratories).

### Screening of the drug library

Each compound in our library consisting of 1,165 drugs was provided by pharmaceutical companies. RAW264.7 macrophages were cultured for 24 h in the absence (vehicle) or presence of each drug at 10 µM, and CD9 and CD81 protein expressions relative to actin were quantified by immunoblotting and densitometry analysis. Drugs that increased the level of CD9 or CD81 more than 1.5-fold relative to vehicle alone were regarded as positive. False positive drugs were excluded by visual inspection of the blots.

### Cell culture, statin treatment, and LPS stimulation

The mouse macrophage line RAW264.7, the human monocytic cell line THP-1, and the mouse fibroblast cell line NIH3T3 were cultured in DMEM containing 10% heat-inactivated FBS, 100 U/ml penicillin, and 100 µg/ml streptomycin. Mouse bone marrow-derived macrophages (BMDMs) were prepared as previously described [15]. Briefly, cells were isolated by flushing tibia and femur bone marrow and cultured in DMEM supplemented with 20% FBS and 30% L929 supernatant containing macrophage-stimulating factor. After 4-6 days of culture, adherent macrophages were detached and resuspended in DMEM supplemented with 10% FBS, 100 U/ml penicillin, and 100 µg/ml streptomycin. Phenol-extracted LPS from *Escherichia coli* O55:B5 was purchased from Sigma-Aldrich. RAW264.7 cells or BMDMs were either not treated or treated for 24 h with 0.1-10 µM fluvastatin or simvastatin in culture medium containing 0.02% BSA, and then either not stimulated or stimulated by adding 0.1 or 1 µg/ml LPS. In some experiments, RAW264.7 cells were treated with 1 mM mevalonolactone (mevalonate), 100 µM farnesyl pyrophosphate (FPP), 100 µM geranylgeranyl pyrophosphate (GGPP), 1 mM squalene, 20 µM farnesyl transferase inhibitor (FTI-277), 20 µM geranylgeranyl transferase inhibitor (GGTI-298), or Rho kinase inhibitor (HA-1077), all of which were purchased from Sigma-Aldrich, and cultured in the absence or presence of 10 µM fluvastatin or simvastatin or 30 µM zoledrontate. In other experiments, RAW264.7 cells were treated with theophylline or dexamethasone in the presence of 10 or 50 ng/ml TSA (Wako Pure Chemical Industries) as previously described [12].

### Flow cytometry

After Fc receptors were blocked with anti-CD16/CD32 mAb (BD Biosciences), RAW264.7 cells were labeled with rat biotinylated anti-mouse CD9 mAb (KMC8), hamster biotinylated anti-mouse CD81 mAb (Eat2), rabbit biotinylated anti-LAMP3 (CD63) polyclonal Ab, and rat biotinylated anti-mouse integrin β1 subunit mAb (KMI6, BD Biosciences), and then stained with FITC-conjugated streptavidin. Stained cells were analyzed on a BD FACSCanto II (BD Biosciences).

### Reverse transcription and real-time PCR

Total RNA was extracted with Isogen (Nippon Gene), and 1 µg RNA was subjected to RT-PCR amplification using the following oligonucleotide primers: *CD9*, 5′-CCTCCCTCAGGAGTGTACATTC-3′ and 5′-GAGGAACCCGAAGAACTAGAAGAC-3′; *CD81*, 5′-TGTGAGGTGGCTGCAGGCATCTGG-3′ and 5′-TCTCATGGAAAGTCTTCACCACAG-3′. The thermal-cycling parameters were 30 cycles of 30 s at 94°C, 30 s at 60°C, and 60 s at 72°C; we confirmed that these parameters yielded the amplification of template DNAs within a linear range. Quantitative real-time PCR was carried out using an ABI PRISM 7900HT Detection System with TaqMan Universal PCR Master Mix and primers for CD9 (ID: Mm00514275_g1) and CD81 (ID: Mm00504869_m1) (Applied Biosystems) according to the manufacturer’s instructions. Relative expression levels of tetraspanin gene were assessed from ΔCt values, taking efficiency values into account, and normalizing to the levels of *GAPDH*.

### Cytokine analysis, gelatin zymography, and cell spreading assay

Concentrations of TNF-α in culture supernatants or bronchoalveolar lavage fluid (BALF) were measured by ELISA using Quantikine (R&D Systems). Samples containing equal amounts of protein from culture supernatants or BALF were electrophoresed on a 10% polyacrylamide gel containing 0.1% gelatin (Invitrogen). Gels were washed with 2.5% Triton X-100 and incubated at 37°C overnight in Novex zymogram developing buffer (Invitrogen). Gelatinolytic bands were visualized by staining with 0.1% Coomassie Brilliant Blue R250. The intensity of the lytic bands was quantified on a FluorChem (ProteinSimple). For the spreading assay, cells were visualized using Giemsa stain and photographed. Percentages of spread cells were determined according to their longest diameters.

### Sucrose gradients

RAW264.7 cells were lysed for 1 h on ice in 500 µl of MES buffer (150 mM NaCl and 20 mM MES [pH 6.5]) supplemented with 1% Triton X-100, 2 mM PMSF, 10 µg/ml aprotinin, and 10 µg/ml leupeptin. Lysates were then sheared by successive passage through hypodermic needles (5 × 18G11/2, 10 × 26G1/2). The lysate was mixed with an equal volume of 90% sucrose in MES buffer, placed at the bottom of a centrifuge tube, and overlaid with 4.5 ml of 30% sucrose and 3.5 ml of 5% sucrose in MES buffer. After centrifugation at 100,000 × *g* for 16.5 h at 4°C in a Beckman SW40Ti rotor, 1-ml fractions were collected from the top of the gradient. Each fraction was added with *n*-octylglucoside (60 µM final) and analyzed by SDS-PAGE using 5-20% gradient gels (Wako Pure Chemical Industries). Protein distribution in the fractions was visualized by immunoblotting with anti-CD14, anti-CD9, anti-CD81, anti-flotillin-1, and anti-CD45 Abs. The density of blots was quantified on a FluorChem. In some experiments, low-density (lipid-enriched) light membrane fractions or dense fractions were pooled and subjected to immunoprecipitation using anti-CD14 mAb. Immune complexes were subjected to SDS-PAGE and probed with biotinylated anti-CD9 or anti-CD81 mAbs, followed by peroxidase-conjugated streptavidin.

### Statin treatment and LPS challenge *in vivo*

Mice were intraperitoneally injected with 20 or 30 mg/kg fluvastatin or simvastatin 24 h, 12 h, or 1 h before or 12 h or 24 h after LPS challenge. LPS was administered intranasally at 0.5 mg/kg to anesthetized mice. Between 2 h and 4 days later, bronchoalveolar lavage was performed, or histological lung sections were prepared and inspected. In other experiments, LPS was intraperitoneally administered to mice at 30 or 40 mg/kg and survival was monitored.

### Bronchoalveolar lavage and histology

Lungs of anesthetized mice were subjected to lavage with three volumes of 1 ml PBS containing 0.1% BSA. Collected cells in the BALF were centrifuged onto Cytospin slides and visualized using Diff-Quick stain. Total cell counts were determined using a hemocytometer. The supernatants of BALF samples containing an equal amount of protein were subjected to TNF-α measurement and gelatin zymography. For histology, lungs were inflated to 25 cm water pressure with 10% buffered neutral formalin via an intratracheal cannula and then embedded in paraffin. Parasagital 5-µm-thick sections were stained with hematoxylin and eosin.

### Statistical analysis

*In vitro* assays were performed on quadruplicate cultures. Animal experiments were performed using at least four mice in each group. All numerical results are expressed as mean ± SEM. Statistical differences were determined by two-tailed Student’s *t* test. *P* < 0.05 was considered statistically significant. Significance in survival of LPS-challenged mice was determined by Kaplan-Meier estimates and the log-rank test.

## Results

### Screen of a drug library for agents that upregulate CD9 or CD81 in RAW264.7 macrophages

**Figure 1:**
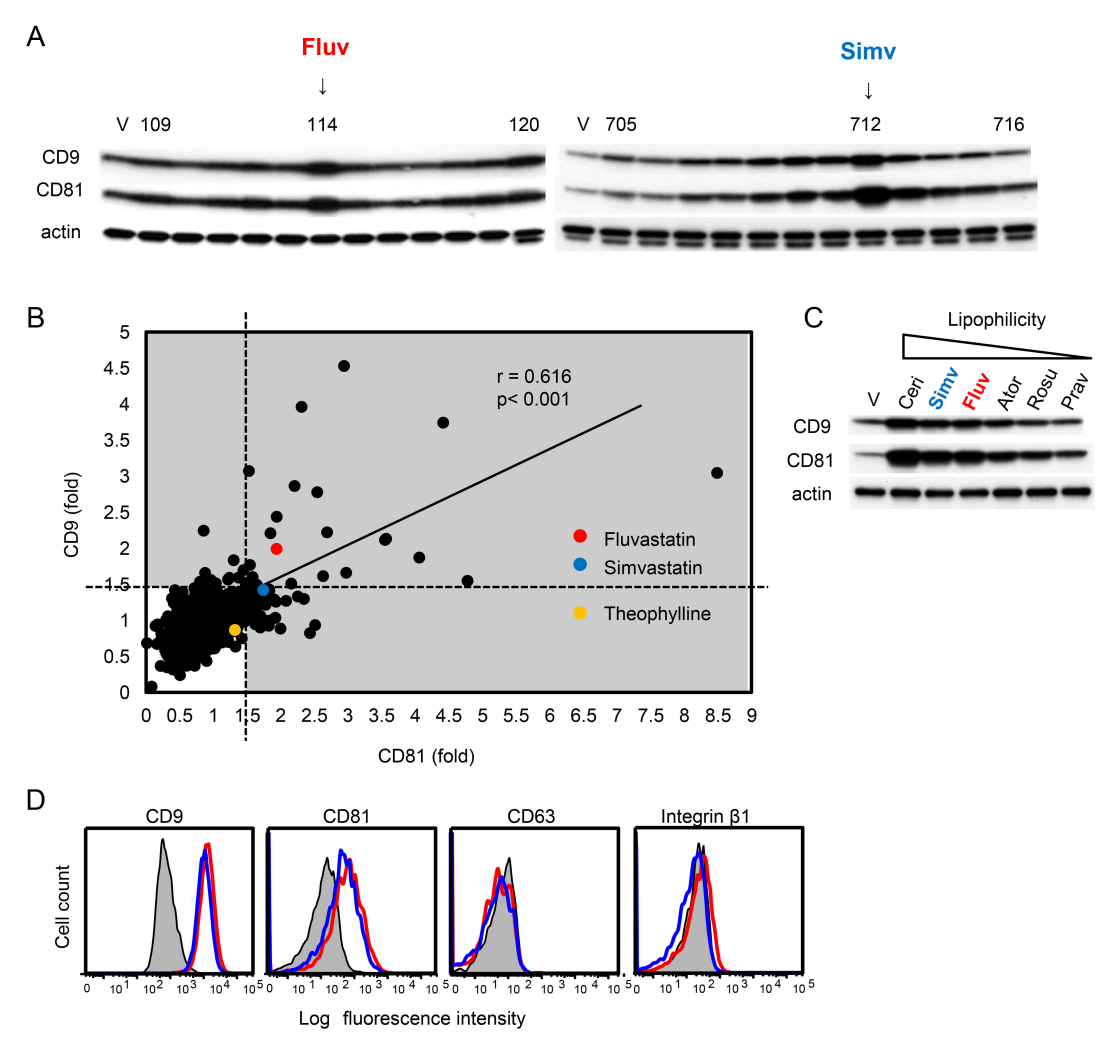
Screening of a drug library for agents that upregulate CD9 or CD81 in RAW264.7 macrophages. (**A**) RAW264.7 cells were cultured for 24 h in the absence (V, vehicle alone) and presence of each drug (10 µM). The cells were lysed, and levels of CD9 and CD81 were examined by immunoblotting. Blots of results with fluvastatin (Fluv) and simvastatin (Simv) are shown. Anti-actin blots show that comparable amounts of protein were loaded in each lane. (**B**) After testing 1,165 drugs, levels of CD9 and CD81 relative to actin were quantified by densitometry. Fold changes of the expression levels compared with vehicle alone were calculated and plotted. Drugs that increased the level of either CD9 or CD81 more than 1.5-fold compared with vehicle alone were regarded as positive. Correlation between fold changes in CD9 and CD81 levels was analyzed using Pearson’s correlation coefficient. (**C**) RAW264.7 cells were cultured in the absence (V) or presence of multiple statins (10 µM) and levels of CD9 and CD81 were examined by immunoblotting. The statins are arranged in order of decreasing lipophilicity. Ceri, cerivastatin; Simv, simvastatin; Fluv, fluvastatin; Ator, atorvastatin; Rosu, rosuvastatin; Prav, pravastatin. (**D**) RAW264.7 cells were cultured in the absence (shaded histograms) or presence (10 µM) of fluvastatin (open red histograms) and simvastatin (open blue histograms). Surface levels of CD9, CD63, CD81, and the integrin β1 subunit were analyzed by flow cytometry.

Because COPD is a systemic disease complicated with comorbidities in organs other than the lung [3], we speculated that drugs used to treat such comorbidities might include agents that increase levels of macrophage CD9 and/or CD81 and would therefore be effective for COPD. To test this idea, we screened a library of 1,165 drugs currently used in clinics. Mouse RAW264.7 macrophages were cultured in the presence of each drug and changes in the levels of CD9 and CD81 relative to vehicle alone were tested by immunoblotting (Figure 1A) and densitometry analysis (Figure 1B). Fold changes in CD9 level were positively correlated with those of CD81 (*R* = 0.616, *P* < 0.001). Table S1 lists drugs that increased either CD9 or CD81 level more than 1.5-fold compared to vehicle. These 44 screen-positive agents included antidepressants, antiarrhythmic agents, statins, sulfonylureas, antitumor agents, antibiotics, antiparasitic agents, and antiseptics. Among these, we decided to further investigate fluvastatin and simvastatin, because they are not currently used for the treatment of COPD, but are effective against its age-related comorbidities including cardiovascular diseases and perhaps also osteoporosis [16]. Moreover, statins have increasingly been recognized to have anti-inflammatory effects that may be independent of their effects on serum cholesterol [17,18]. Although two other statins, pravastatin and rosuvastatin, were included in the drug library, neither was positive in the screen. When we reassessed multiple statins including cerivastatin, which has been withdrawn from the market worldwide [19], we found that their lipophilicity is important; lipophilic statins had stronger effects than hydrophilic statins (Figure 1C). Flow cytometry confirmed that fluvastatin and simvastatin upregulate surface expression levels of CD9 and CD81 in RAW264.7 macrophages, but not levels of another tetraspanin, CD63, or the integrin β1 subunit (Figure 1D). In agreement with our previous report [12], the HDAC activator theophylline [20] modestly increased the level of CD81 (1.32-fold) in the screen (Figure 1B).

### Fluvastatin and Simvastatin Reverse Downregulation of CD9 and CD81 in LPS-Stimulated RAW264.7 Cells

**Figure 2:**
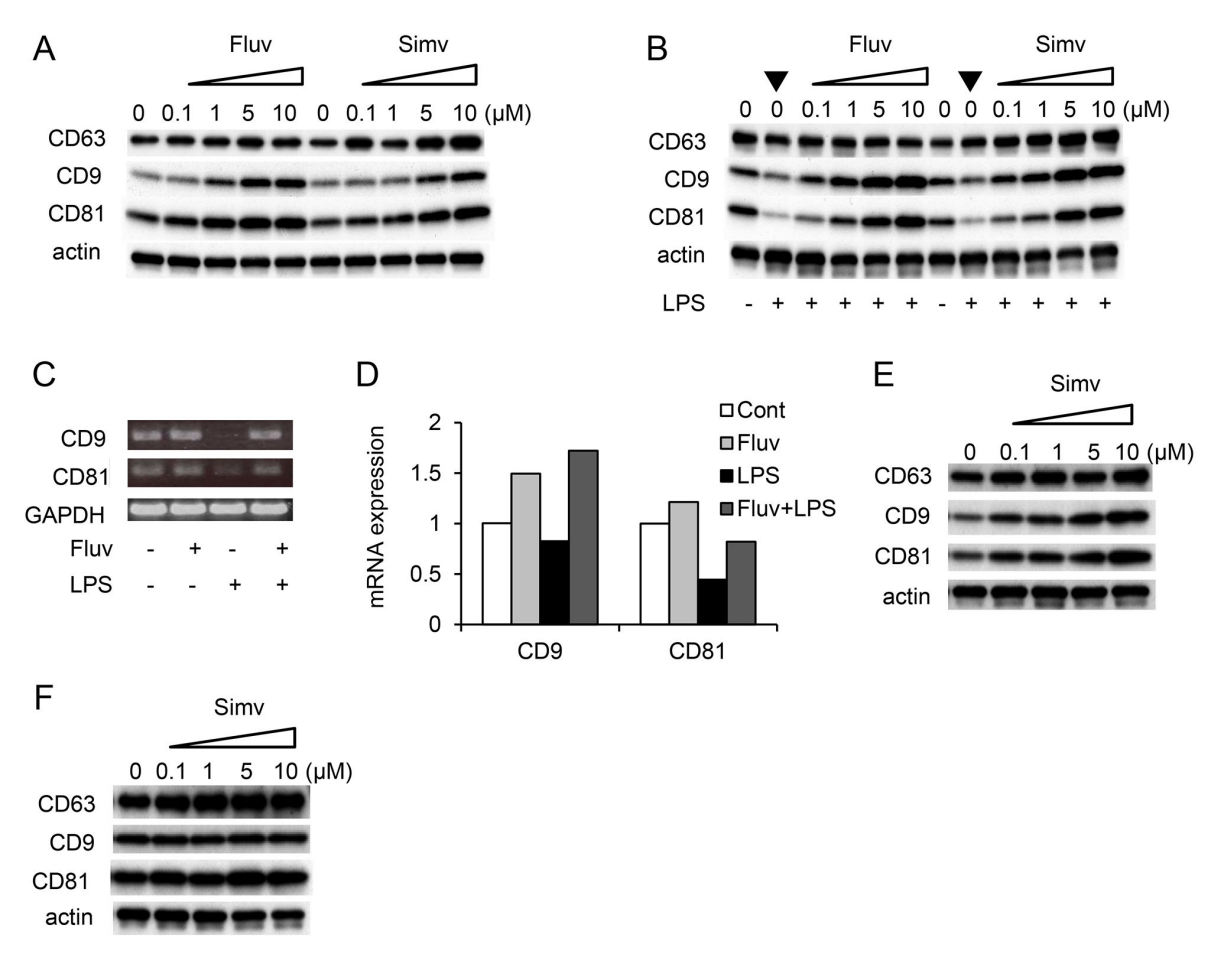
Fluvastatin and simvastatin increase CD9 and CD81 levels in RAW264.7 cells. (**A**) RAW264.7 cells were cultured for 24 h in the absence or presence of increasing concentrations of fluvastatin (Fluv) or simvastatin (Simv). The cells were lysed, and levels of CD9, CD63, and CD81 were examined by immunoblotting. Anti-actin blots show that comparable amounts of protein were loaded in each lane. (**B**) RAW264.7 cells were untreated (-) or cultured in the absence or presence of increasing concentrations of fluvastatin or simvastatin and stimulated for 24 h with 0.1 µg/ml LPS (+). Levels of CD9, CD63, and CD81 were examined by immunoblotting. Note that LPS downregulates CD9 and CD81 in the absence of statins (arrowheads). (**C**) RAW264.7 cells were cultured in the absence (-) or presence of 3 µM fluvastatin (+), and unstimulated (-) or stimulated for 24 h with 1 µg/ml LPS (+). mRNA levels of *CD9* and *CD81* were examined by reverse transcription PCR. *GAPDH* is an internal loading control. (**D**) RAW264.7 cells were cultured in the absence or presence of fluvastatin, and unstimulated or stimulated with LPS. Control (Cont) was an untreated culture. mRNA levels of *CD9* and *CD81* were examined by real-time PCR. Data shown are from one representative of three similar experiments. (**E**) Human monocytic THP-1 cells were treated for 4 h with 1 µg/ml phorbol 12-myristate 13-acetate, allowed to attach to a plate, and then cultured in the absence or presence of increasing concentrations of simvastatin. Levels of CD9, CD63, and CD81 were examined by immunoblotting. (**F**) Mouse 3T3 fibroblasts were cultured in the absence or presence of increasing concentrations of simvastatin. Levels of CD9, CD63, and CD81 were examined by immunoblotting.

To further examine the upregulation of CD9/CD81 by fluvastatin and simvastatin, we cultured RAW264.7 cells in the presence of increasing concentrations of these statins. In unstimulated RAW264.7 cells, CD9 and CD81 levels increased in a dose-dependent manner, but the levels of integrin β1 subunit (data not shown) and CD63 did not change (Figure 2A). When RAW264.7 cells were stimulated with LPS, CD9 and CD81, but not CD63, were downregulated as we previously reported [10]. Fluvastatin and simvastatin prevented this downregulation and increased CD9 and CD81 (Figure 2B) as effectively as in unstimulated cells. Reverse transcription PCR (Figure 2C) and real-time PCR (Figure 2D) revealed that LPS inhibited production of CD9 and CD81, and fluvastatin reversed this inhibition. In the human monocytic cell line THP-1, CD9 and CD81 were also upregulated in a dose-dependent manner by fluvastain (data not shown) and simvastatin (Figure 2E). Meanwhile, these statins did not increase levels of CD9 or CD81 in mouse 3T3 fibroblasts (Figure 2F), mouse primary fibroblasts, or human umbilical vein endothelial cells (data not shown).

### Fluvastatin and simvastatin prevent production of TNF-α and MMP-9 and cell spreading in LPS-stimulated RAW264.7

**Figure 3:**
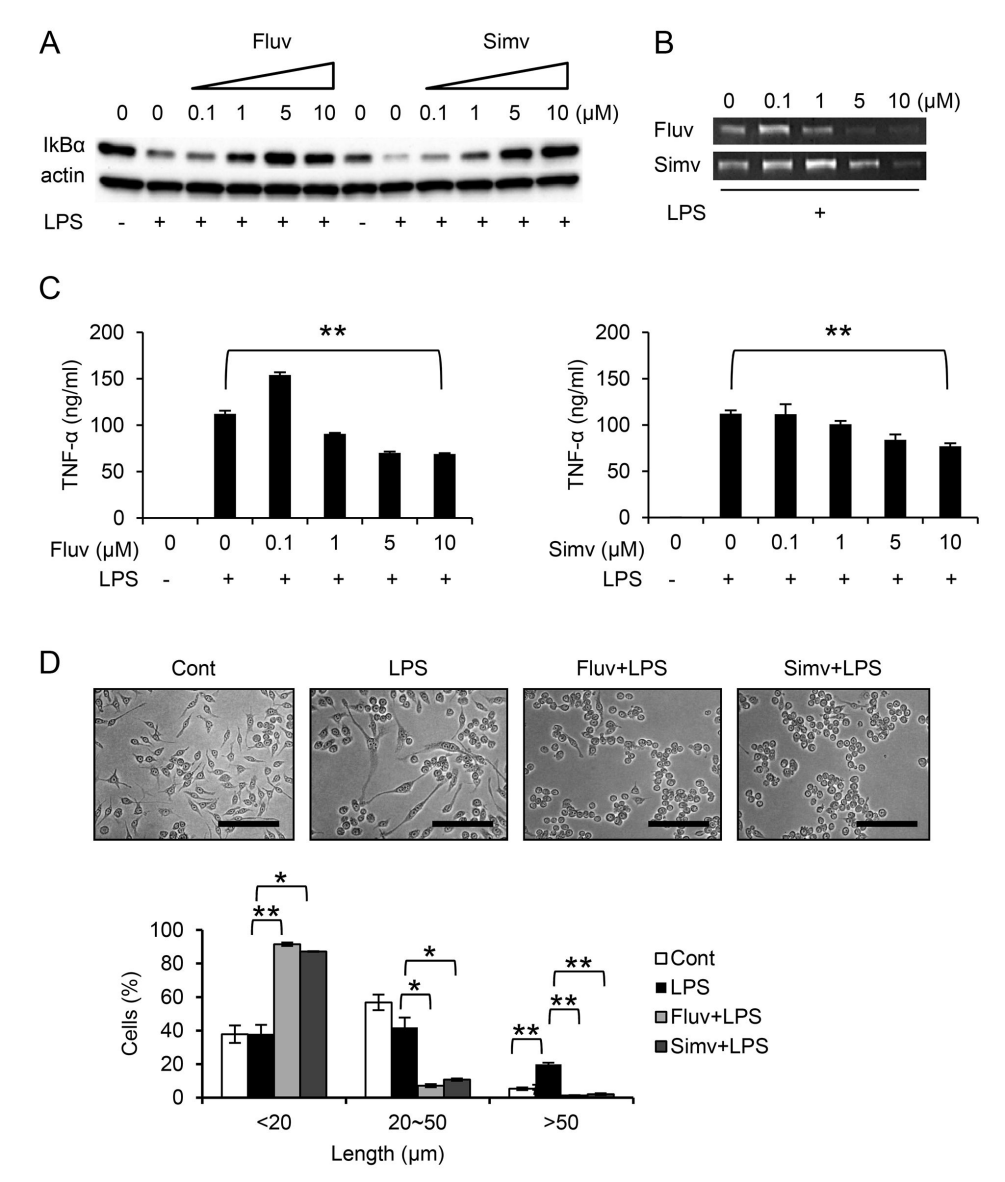
Fluvastatin and simvastatin prevent TNF-α and MMP-9 production and cell spreading in LPS-stimulated RAW264.7. (**A**) RAW264.7 cells were untreated (-) or cultured for 24 h in the absence or presence of increasing concentrations of fluvastatin (Fluv) or simvastatin (Simv) and stimulated for 15 min with 1 µg/ml LPS (+). The cells were lysed and levels of IκBα were examined by immunoblotting. Anti-actin blots show that comparable amounts of protein were loaded in each lane. (**B**) RAW264.7 cells were cultured in the absence or presence of increasing concentrations of fluvastatin (top) or simvastatin (bottom), and stimulated for 5 h with 0.1 µg/ml LPS (+). Activities of MMP-9 in culture supernatants were analyzed by gelatin zymography. (**C**) RAW264.7 cells were untreated (-) or cultured in the absence or presence of increasing concentrations of fluvastatin (left) or simvastatin (right) and stimulated for 5 h with 0.1 µg/ml LPS (+). Concentrations of TNF-α in culture supernatants were measured by ELISA. (**D**) RAW264.7 cells were untreated (Cont, control) or cultured in the absence or presence of 5 µM fluvastatin or simvastatin and stimulated for 4 h with 0.1 µg/ml LPS, and then stained and photographed (upper panel). Scale bar, 100 µm. Percentages of spread cells were determined according to their longest diameters (lower panel). Each bar represents the mean ± SEM. ^⋆^ *P* < 0.05; ^⋆ ⋆^ *P* < 0.01.

When RAW264.7 cells are stimulated with LPS, signals downstream of the CD14/TLR4 receptor complex activate the NF-κB-dependent inflammatory response through degradation of IκB [21]. In addition to their cholesterol-lowering effect, statins exert anti-inflammatory effects. For example, it was reported that simvastatin inhibits production of pro-inflammatory mediators in LPS-stimulated monocytes/macrophages [22,23]. In agreement with these previous reports, fluvastatin and simvastatin dose-dependently prevented the degradation of IκBα in RAW264.7 cells (Figure 3A). This effect was concomitant with prevention of the inflammatory response; the statins dose-dependently suppressed production of MMP-9 (Figure 3B) and TNF-α (Figure 3C) in the presence of LPS. Morphologically, RAW264.7 cells stimulated with LPS exhibited enhanced cell spreading and extended long projections (Figure 3D), as we previously reported [10]. Fluvastatin and simvastatin inhibited this morphologic activation; most statin-treated cells displayed rounded morphologies, although they were still adherent. The statins also prevented cell spreading in the absence of LPS (Figure S1), suggesting that they exert an LPS signal-independent effect on cytoskeletal reorganization. Together, these observations indicate that fluvastatin and simvastatin upregulate IκBα and prevent LPS-induced activation of RAW264.7 macrophages, simultaneously increasing CD9 and CD81 levels.

### Statin treatment results in transfer of CD14 from the lipid raft into the CD9-enriched microdomains

**Figure 4:**
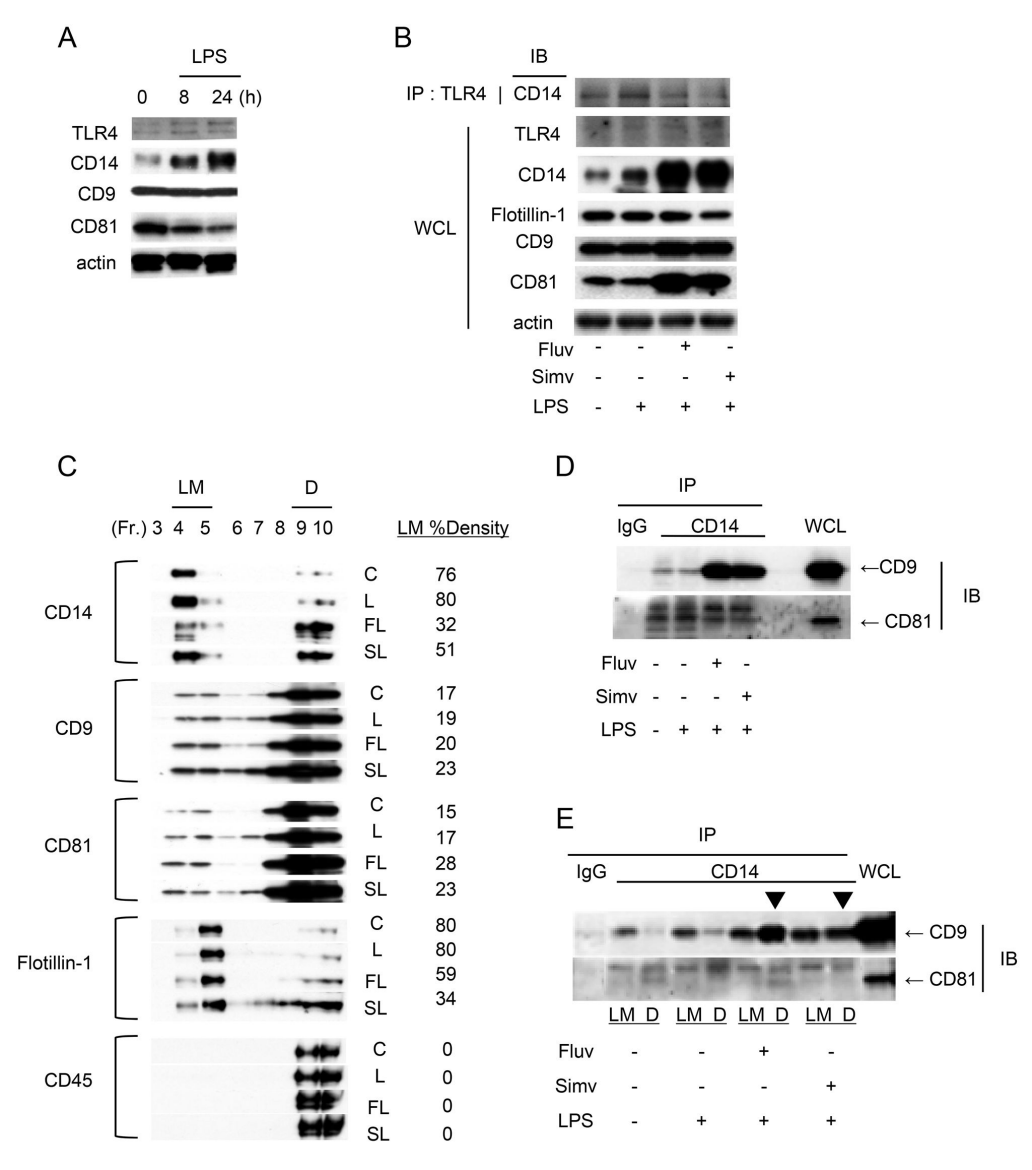
Statins transfer CD14 from lipid rafts into CD9-enriched microdomains. (**A**) RAW264.7 cells were stimulated with 0.1 µg/ml LPS and, after the indicated times, the cells were lysed and protein levels were examined by immunoblotting. Anti-actin blots show that comparable amounts of protein were loaded in each lane. (**B**) RAW264.7 cells were untreated (-) or cultured for 24 h in the absence (-) or presence of 5 µM fluvastatin (Fluv) or simvastatin (Simv) (+) and stimulated for 2 h with 1 µg/ml LPS (+). Proteins in whole-cell lysate (WCL) and CD14 protein in immunoprecipitates (IP) with anti-TLR4 Ab were immunoblotted (IB). (**C**) RAW264.7 cells were treated as in **B**. Lysates of untreated (C, control) cultures or LPS-stimulated cultures in the absence (L) or presence of fluvastatin (FL) or simvastatin (SL) were fractionated by sucrose density gradients, and protein distributions were visualized by immunoblotting. The intensities of blots were quantified by densitometry, and percentages of density units of light membrane (LM) fractions are displayed to the right of the blots. Data shown are from one representative of three similar experiments. (**D**) Immunoblots of CD9 and CD81 proteins in whole-cell lysates and in immunoprecipitates with control IgG or anti-CD14 mAb. (**E**) Immunoblots of CD9 and CD81 proteins in whole-cell lysates and in immunoprecipitates with control IgG or anti-CD14 mAb from pooled LM fractions (4 and 5) and dense (D) fractions (9 and 10). In the presence of statins, more CD14/CD9 complexes were formed in dense fractions (arrowheads).

As the binding site for LPS, the GPI-linked protein CD14 sits at the apex of all cellular responses to LPS and functions to induce an innate immune cascade [24]. In naive macrophages, a large fraction of CD14 on the membrane resides in lipid rafts; upon stimulation with LPS, even more CD14 protein concentrates into rafts to form the LPS recepor clusters, which contain other signaling molecules, including TLR4 [25]. We previously reported that CD9 associates with CD14 and prevents formation of the CD14/TLR4 receptor complex in macrophages [10]. To explore the involvement of CD9 and CD81 in the anti-inflammatory effect of statins, we studied the expressions of CD14, CD9, and CD81, as well as the associations between these proteins. When RAW264.7 cells were stimulated with LPS, CD14 and TLR4 were upregulated, whereas CD9 and CD81 were downregulated, as revealed in an experiment using whole-cell lysates (Figure 4A). This was in contrast to cultures without LPS stimulation, in which levels of CD9 and CD81 were not changed, and CD14 and TLR4 levels decreased over time (Figure S2A). The addition of fluvastatin or simvastatin to LPS-stimulated cultures increased levels of CD9 and CD81, whereas it did not significantly change levels of TLR4 or the raft-marker protein flotillin-1 (Figure 4B). CD14, which was already upregulated by LPS, was further increased by the statins, possibly due to a reduction in the level of CD14’s soluble form and an increase in the level of membrane-bound form; both forms participate in cellular signaling, as reported in lovastatin-treated RAW264.7 cells [26]. LPS increased levels of CD14/TLR4 receptor complexes, and statin treatment decreased complex formation (Figure 4B, top panel), accounting for the decrease in inflammatory signaling.

Next, we examined subcellular compartmentalization by sucrose gradient centrifugation (Figure 4C). Following LPS stimulation, CD14 protein was concentrated in low-density light membrane fractions corresponding to lipid rafts, and notably, addition of statins reverse-transferred CD14 from rafts to dense (non-raft) fractions. The distribution of flotillin-1 was likewise shifted from raft to non-raft fractions by the statins. By contrast in the same gradients, CD9 and CD81 proteins were mostly localized in dense fractions, and the addition of statins rather increased the proportion of these proteins present in light membrane fractions, calculated as the percentage of total density units (Figure 4C). The distribution of the nonraft protein CD45 in dense fractions was not affected by addition of LPS or statins. In another experiment, the shift of CD14 towards dense fractions was reproduced; again, the distribution of CD14 was different from that of CD9, CD81, and integrin β2 subunit (Figure S2B). In our previous report, we showed in coprecipitation experiments that CD14 associates with CD9 in light membrane fractions regardless of LPS stimulation [10]. To examine the effects of statins on CD14/CD9 complex formation, we performed similar coprecipitation experiments using whole-cell lysate (Figure 4D) and pooled light membrane fractions and dense fractions (Figure 4E). As shown in Figure 4D, upper panel, fluvastatin and simvastatin dramatically increased the association between CD14 and CD9. This increase was not solely due to the increased level of CD14, because LPS alone did not increase the association despite upregulating CD14 (Figure 4B); furthermore, the increase was specific, because the association between CD14 and the integrin β2 subunit [27] was not increased by fluvastatin (Figure S2C). Importantly, the CD14/CD9 complex formation occurred in dense fractions rather than light membrane fractions in the presence of statins (Figure 4E, upper panel). Although the statins upregulated CD81, CD14/CD81 complex formation was minimal compared with CD14/CD9 complex formation, and was not increased by the statins (Figure 4, D and E, lower panels). These results suggest that fluvastatin and simvastatin transfer CD14 from lipid rafts into non-raft CD9-enriched microdomains, thereby preventing LPS receptor formation in rafts.

### Anti-inflammatory effects of statins are CD9-dependent

**Figure 5:**
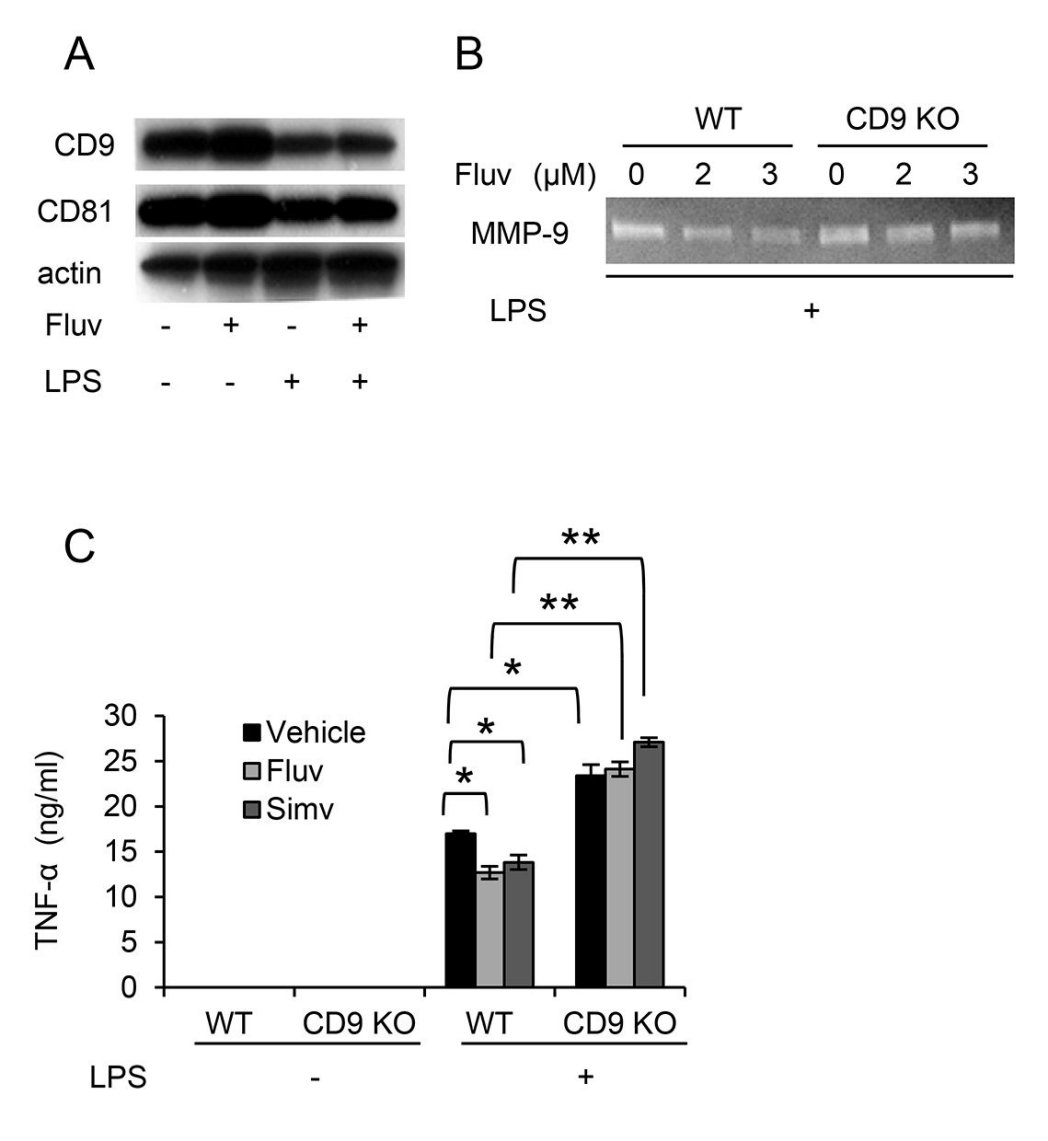
The anti-inflammatory effects of statins are CD9-dependent. (**A**) BMDMs from WT mice were cultured for 24 h in the absence (-) or presence of 3 µM fluvastatin (Fluv) (+), and unstimulated (-) or stimulated for 24 h with 1 µg/ml LPS (+). The cells were lysed, and levels of CD9 and CD81 were examined by immunoblotting. Anti-actin blots show that comparable amounts of protein were loaded in each lane. (**B**) BMDMs from WT and CD9 KO mice were cultured in the absence or presence of the indicated concentrations of fluvastatin, and stimulated for 18 h with 10 µg/ml LPS (+). Activities of MMP-9 in culture supernatants were analyzed by gelatin zymography. (**C**) BMDMs from WT and CD9 KO mice were cultured in the absence (vehicle) or presence of 10 µM fluvastatin or simvastatin (Simv), and unstimulated (-) or stimulated for 18 h with 1 µg/ml LPS (+). Concentrations of TNF-α in culture supernatants were measured by ELISA. Each bar represents the mean ± SEM. ^⋆^ *P* < 0.05; ^⋆ ⋆^ *P* < 0.01.

**Figure 6:**
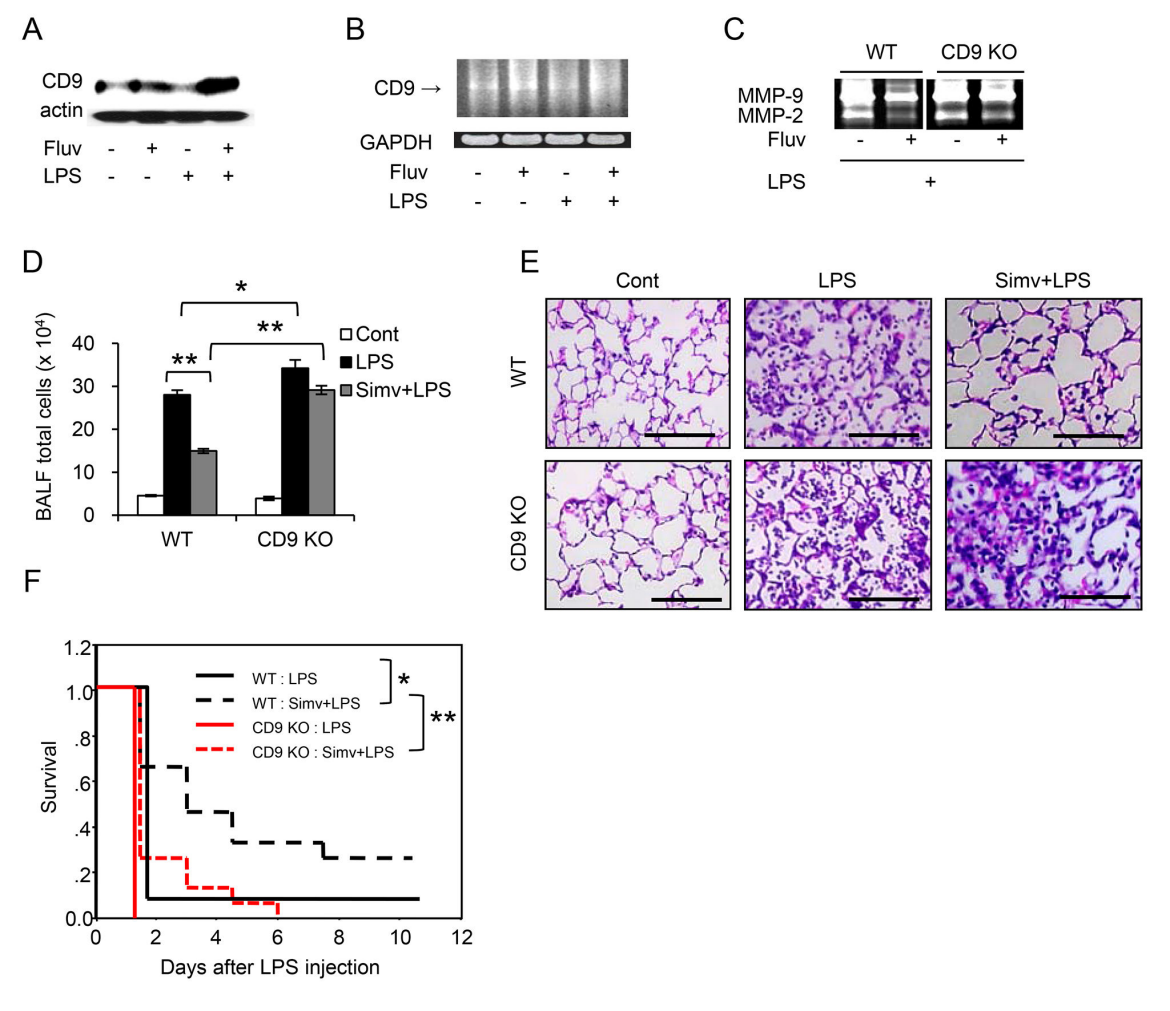
Statins protect mice from LPS-induced injury in a CD9-dependent manner. (**A**) WT mice were repeatedly intraperitoneally injected with vehicle (-) or 30 mg/kg fluvastatin (Fluv), and unchallenged (-) or intraperitoneally challenged with 30 mg/kg LPS (+). After 48 h, BMDMs were isolated and the level of CD9 was examined by immunoblotting. Anti-actin blots show that comparable amounts of protein were loaded in each lane. (**B**) WT mice were treated as in **A**. *CD9* mRNA levels in the BMDMs were examined by reverse transcription PCR. *GAPDH* is an internal loading control. (**C**) WT and CD9 KO mice were repeatedly intraperitoneally injected with vehicle (-) or 30 mg/kg fluvastatin (+), and intranasally challenged with 0.5 mg/kg LPS (+). After 24 h, activities of MMP-2 and MMP-9 in BALF were analyzed by gelatin zymography. (**D**) WT and CD9 KO mice were untreated (Cont, control) or intraperitoneally injected with vehicle or 20 mg/kg simvastatin (Simv) and intranasally challenged with 0.5 mg/kg LPS. After 4 days, total cells in BALF from the mice from each group (*n* = 9) were counted using a hemocytometer. Each bar represents the mean ± SEM. (**E**) WT and CD9 KO mice were treated as in **D**. Histological lung sections collected at 4 days were stained with hematoxylin and eosin. Scale bar, 100 µm. (**F**) WT and CD9 KO mice were intraperitoneally injected with vehicle or 20 mg/kg simvastatin, and intraperitoneally challenged with 40 mg/kg LPS. Survival of the mice from each group (*n* = 12) was monitored and analyzed by the Kaplan-Meier method. ^⋆^ *P* < 0.05; ^⋆ ⋆^ *P* < 0.01.

To determine whether CD9 is required for the anti-inflammatory effects of statins, we isolated bone marrow-derived macrophages (BMDMs) from WT and CD9 KO mice, and examined the effects of statins on these cells. Fluvastatin upregulated CD9 and CD81 in untreated and LPS-treated WT BMDMs (Figure 5A), as it did in RAW264.7 cells. We next tested production of inflammatory mediators in BMDMs stimulated with LPS. As shown in Figure 5B, fluvastatin suppressed LPS-induced production of MMP-9 in WT BMDMs, but the degree of suppression was lower in CD9 KO BMDMs. CD9 KO BMDMs produced more TNF-α than WT BMDMs did in response to LPS, as we previously reported [10], and fluvastatin and simvastatin significantly inhibited the production of TNF-α in WT BMDMs, but not in CD9 KO BMDMs (Figure 5C). To examine CD9-dependent anti-inflammatory effects of statins *in vivo*, we treated CD9 KO mice and WT littermates with statins and compared the effects. WT mice were repeatedly injected with fluvastatin and intraperitoneally challenged with LPS, and then BMDMs were isolated and examined for the expression of CD9. Consistent with the results of *in vitro* experiments, CD9 was upregulated by fluvastatin at the protein (Figure 6A) and mRNA level (Figure 6B), regardless of the LPS challenge. Next, we administered LPS intranasally to WT and CD9 KO mice, and evaluated MMP activities in the lung by gelatin zymography of bronchoalveolar lavage fluid (BALF). As shown in Figure 6C, treatment with fluvastatin suppressed gelatinolytic activities of MMP-9 and MMP-2 in WT mice but suppression was not obvious in CD9 KO mice. We also measured TNF-α levels; fluvastatin decreased LPS-induced TNF-α production in BALF of WT mice (1.52 ± 0.38 ng/ml with vehicle *vs.* 1.18 ± 0.25 ng/ml with fluvastatin) but not in BALF of CD9 KO mice (1.63 ± 0.19 ng/ml with vehicle *vs.* 1.90 ± 0.45 ng/ml with fluvastatin), although this difference was not statistically significant (*n* = 4). Because simvastatin has been used more frequently in animal models [28,29,30], we performed additional experiments after treatment with simvastatin. LPS was intranasally administered to WT and CD9 KO mice that had been injected with simvastain, and BALF or histological lung sections were analyzed 4 days later. Although neutrophil influx is observed earlier, the predominant cells infiltrating into the lung at this phase are macrophages [31]. BALF cell count revealed that more inflammatory cells infiltrated into the lung in CD9 KO mice than in WT mice, indicating enhanced lung inflammation occurring in CD9 KO mice, as observed in our previous study [10]. Simvastatin prevented cell infiltration in WT mice, but not in CD9 KO mice (Figure 6D). Lung histology also revealed that simvastatin decreased LPS-macrophage infiltration in WT mice but not in CD9 KO mice (Figure 6E). We also examined survival of mice after intraperitoneal LPS injection. Simvastatin prolonged survival of WT mice, but the survival benefit was not significant in CD9 KO mice (Figure 6F). These results suggest that CD9 is required for the anti-inflammatory effects of statins.

### CD9 and CD81 are upregulated by blockade of the mevalonate pathway

**Figure 7:**
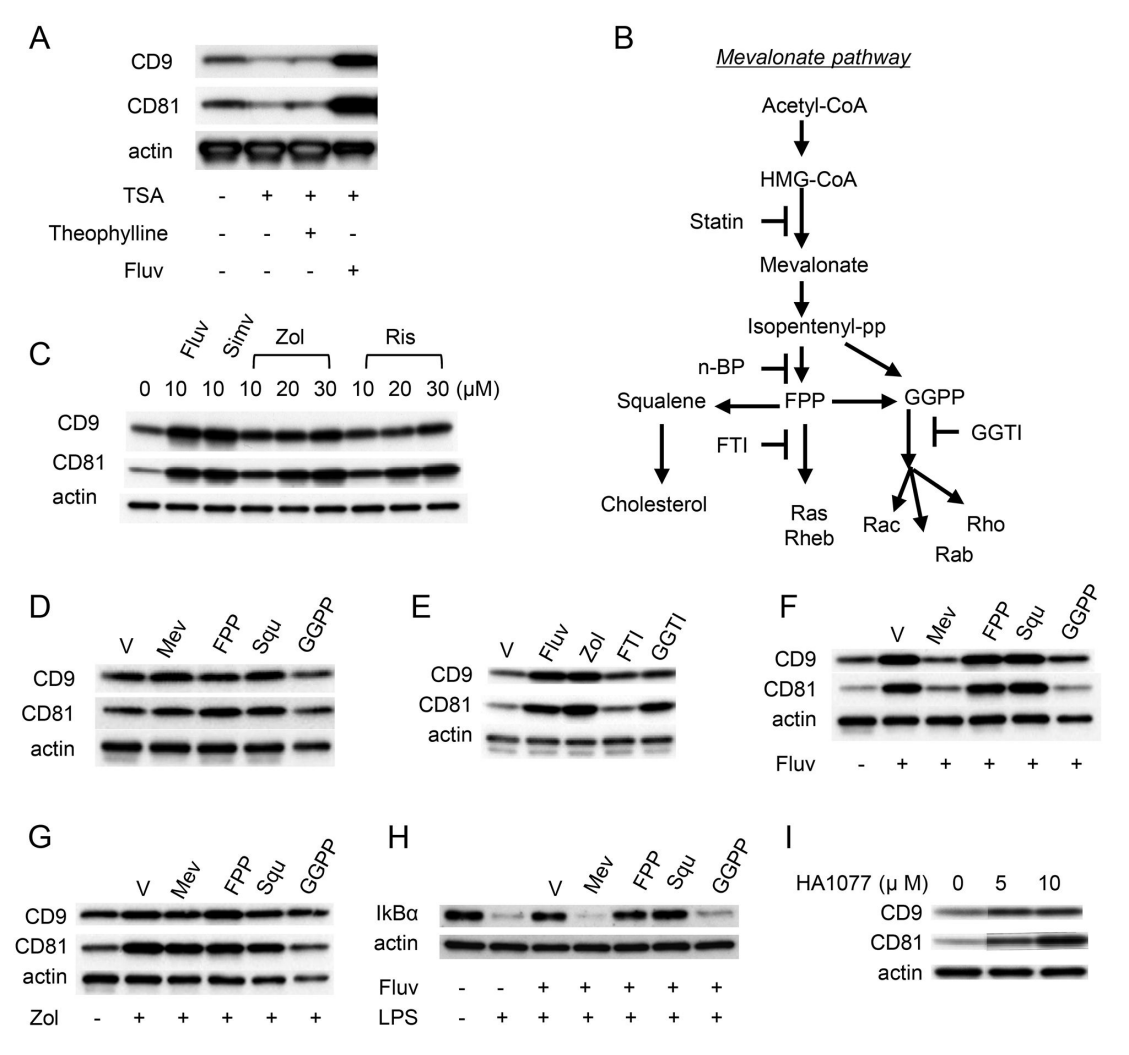
Blockade of the mevalonate pathway increases CD9 and CD81. (**A**) RAW264.7 cells were untreated (-) or treated for 48 h with 50 ng/ml TSA (+) in the absence (-) or presence of 50 µM theophylline or 0.5 µM fluvastatin (Fluv) (+). The cells were lysed, and levels of CD9 and CD81 were examined by immunoblotting. Anti-actin blots show that comparable amounts of protein were loaded in each lane. (**B**) The mevalonate pathway and inhibitors. n-BP, nitrogenous bisphosphonate. (**C**) RAW264.7 cells were cultured for 24 h in the presence of indicated concentrations of fluvastatin, simvastatin (Simv), zoledronate (Zol), or risedronate (Ris). Levels of CD9 and CD81 were examined by immunoblotting. (**D**) RAW264.7 cells were cultured for 24 h in the absence (V, vehicle alone) or presence of mevalonate (Mev), farnesyl pyrophosphate (FPP), squalene (Squ), or geranylgeranyl pyrophosphate (GGPP). Although the actin level in the GGPP lane appears to be lower, an equal amount of protein was loaded. (**E**) RAW264.7 cells were cultured for 24 h in the absence (V) or presence of fluvastatin, zoledronate, farnesyl transferase inhibitor (FTI), or geranylgeranyl transferase inhibitor (GGTI). (**F**) RAW264.7 cells were untreated (-) or treated with fluvastatin (+) in the absence (V) or presence of mevalonate, FPP, squalene, or GGPP. (**G**) RAW264.7 cells were untreated (-) or treated with zoledronate (+) in the absence (V) or presence of mevalonate, FPP, squalene, or GGPP. (**H**) RAW264.7 cells were untreated (-) or treated with fluvastatin (+) in the absence (V) or presence of mevalonate, FPP, squalene, or GGPP and stimulated for 15 min with 0.1 µg/ml LPS (+). The cells were lysed, and levels of IκBα were examined by immunoblotting. (**I**) RAW264.7 cells were cultured for 24 h in the indicated concentrations of HA1077. Levels of CD9 and CD81 were examined by immunoblotting.

We and others have shown that inactivation of HDAC (e.g., by addition of TSA or cigarette smoke extract) lowers CD9 and CD81 levels in RAW264.7 cells [12,13]. Therapeutic concentrations of theophylline and dexamethasone enhance the activity of HDAC [20] and restored the levels of CD9 and CD81 [12] (Figure S3A). To determine whether HDAC activity is involved in the upregulation of CD9/CD81 by statins, we added fluvastatin to RAW264.7 cell culture containing the HDAC inhibitor, TSA. As shown in Figure 7A, CD9 and CD81 were downregulated in the presence of 50 ng/ml TSA, and the addition of theophylline could not reverse this inhibitory effect, although it could do so when 10 ng/ml TSA was used [12] (Figure S3A). Meanwhile, fluvastatin at 0.5 µM abolished this inhibitory effect of TSA, suggesting that the upregulation of CD9 and CD81 is independent of HDAC activity. Because statins are inhibitors of HMG-CoA reductase, we hypothesized that the upregulation of CD9/CD81 may be mediated by blockade of the mevalonate pathway [32]. To test this idea, we treated cells with nitrogenous bisphosphonates, which also block the mevalonate pathway downstream of statins (Figure 7B). Three nitrogenous bisphosphonates (alendronate, risedronate, and zoledronate) included in the drug library were not positive in the first screen (Table S1), but we found that risedronate upregulated CD81 (1.48-fold compared to vehicle) to a greater extent than theophylline (1.32-fold compared to vehicle). We reassessed increasing concentrations of risedronate and zoledronate and observed dose-dependent upregulation of CD9 and CD81 in RAW264.7 cells, although their effects were smaller than those of the statins (Figure 7C). To study the involvement of the mevalonate pathway in more detail, we cultured RAW264.7 cells in the presence of mevalonate and intermediates. Although the addition of mevalonate, farnesyl pyrophosphate (FPP), and squalene did not affect levels of CD9 and CD81, geranylgeranyl pyrophosphate (GGPP) modestly decreased their levels (Figure 7D). In addition, geranylgeranyl transferase inhibitor (GGTI), but not farnesyl transferase inhibitor (FTI), increased CD9 and CD81 to levels comparable to those in cells treated with fluvastatin and zoledronate (Figure 7E), suggesting that mevalonate-dependent post-translational geranylgeranylation of Rho GTPases may lead to downregulation of CD9 and CD81. Consistent with these findings, the upregulation of CD9/CD81 by fluvastatin was abolished by mevalonate or GGPP, but not by FPP or squalene (Figure 7F). Meanwhile, the effect of zoledronate, which acts downstream of mevalonate production, was not abolished by mevalonate (Figure 7G). These results further implicate the mevalonate-GGPP-GTPase branch cascade. In parallel with the CD9/CD81 levels in Figure 7F, the restoration of IκBα level by fluvastatin was suppressed by mevalonate or GGPP, but not by FPP or squalene in LPS-stimulated RAW264.7 (Figure 7H). Experiments using simvastatin yielded similar results. Upregulation of CD9/CD81 (Figure S3B) and increased levels of IκBα (Figure S3C) were abolished by mevalonate but not FPP. Finally, we reasoned that decreased RAW264.7 cell spreading in the presence of statins, which were shown in Figure 3D and Figure S1, might reflect alteration of Rho GTPase signaling [33]. To test this, we exposed cells to a Rho-kinase inhibitor, HA1007. As shown in Figure 7I, inhibition of the Rho-kinase increased levels of CD9 and CD81. Together, these results suggest that the mevalonate-GGPP-Rho pathway negatively regulates the expression of CD9/CD81.

## Discussion

Previous reports including ours suggested that reduction in the levels of tetraspanins CD9 and CD81 may be involved in the progression of inflammatory lung diseases, especially COPD. Several lines of evidence support this idea. First, macrophage CD9 and CD81 are downregulated by LPS, cigarette smoke extract, or the HDAC inhibitor TSA [10,12,13]. LPS is a potent inducer of lung inflammation [21] and is contained in cigarette smoke, which causes pulmonary emphysema. Also, an important mechanism of COPD is inactivation of HDACs (especially HDAC2) by cigarette smoke, resulting in sustained LPS-induced activation of macrophages [1]. LPS itself inactivates HDAC2 by *S*-nitrosylation [34]. Second, when stimulated by LPS, CD9 KO macrophages are strongly activated *in vitro*, and CD9 KO mice develop enhanced lung inflammation, as evidenced by prominent cell spreading and enhanced production of TNF-α and MMPs in alveolar macrophages [10]. Third, CD9/CD81 DKO macrophages are spontaneously activated and produce more MMP-9 than WT macrophages, and CD9/CD81 DKO mice suffer from age-related pulmonary emphysema and osteoporosis, phenotypes akin to human COPD [12,35]. Moreover, we have found that levels of these tetraspanins are decreased in blood monocytes from COPD patients (B Zhou and I Tachibana, unpublished data). These results raise the possibility that upregulation of CD9 and/or CD81 could be used as a novel therapeutic approach.

In this study, in order to identify agents that upregulate macrophage CD9 and CD81, we screened more than 1,000 drugs. Among the positive agents, we focused on the statins, HMG-CoA reductase inhibitors that lower plasma cholesterol by blocking the mevalonate pathway. Statins have been used to prevent and manage cardiovascular diseases, but numerous recent reports have shown that their effects are not limited to cardiovascular diseases, and that these drugs might be used to treat osteoporosis, Alzheimer disease, rheumatoid arthritis, acute lung injury, and COPD [32,36,37]. These pleiotropic effects of statins may be mediated by cholesterol-independent, anti-inflammatory actions, but the precise mechanisms remain unknown [17,18]. The results of this study suggest that tetraspanins, especially CD9, may be an essential player in the anti-inflammatory effects of statins. First, upregulation of CD9 and CD81 was concurrent with the inhibition of inflammatory activation in RAW264.7 macrophages *in vitro*. It has been demonstrated that statins inhibit production of pro-inflammatory mediators in LPS-stimulated RAW264.7 cells [23,38]. This anti-inflammatory effect of statins was dose-dependently reproduced in RAW264.7 as evidenced by the inhibitions of IκB degradation, MMP-9 activity, TNF-α production, and cell spreading (Figure 3), and was paralleled by the upregulations of CD9 and CD81 (Figure 2). Second, statins failed to inhibit LPS-induced activation of CD9 KO BMDMs *in vitro*. CD9 KO BMDMs are strongly activated upon stimulation with LPS as evidenced by increased MMP-9 activity and TNF-α production described in our previous report [10], and in this study these effects were not suppressed by statins in CD9 KO BMDMs, in contrast to the situation in WT BMDMs (Figure 5). Third, statins failed to inhibit LPS-induced lung inflammation and to prolong survival of CD9 KO mice *in vivo*. Simvastatin exerts anti-inflammatory effects on lung inflammation in animal models [28,30] as well as in humans [39,40]. Such *in vivo* anti-inflammatory effects were not observed in CD9 KO mice, but were significant in WT mice (Figure 6). Although we focused on macrophages to study upregulation of CD9/CD81 by the statins, anti-inflammatory effects of statins were also reported in other inflammatory cells including neutrophils and lymphocytes and in structural cells including epithelial cells and smooth muscle cells [17,36]. Thus, we could not exclude effects of the statins on these cell lineages in the *in vivo* experiments of this study.

Using subcellular membrane fractionation of RAW264.7 cells on sucrose gradients, we showed previously that CD9 associates with the LPS signaling mediator CD14; furthermore, in CD9 KO BMDMs, CD14 and TLR4 concentration in lipid rafts and formation of the CD14/TLR4 complex are increased relative to WT BMDMs, suggesting that CD9 prevents the formation of the LPS receptor cluster [10]. By upregulating CD9 in LPS-stimulated macrophages, fluvastatin and simvastatin most likely restore negative regulation of LPS signals by this tetraspanin. One of the pleiotropic effects of statins was reported to be disruption of raft proteins by cholesterol depletion from the plasma membrane [41,42]. Indeed, we observed that CD14 was transferred from light membrane fractions (containing rafts) to dense (non-raft) fractions (Figure 4C and Figure S2B). Such translocation was not observed for CD9 or CD81. Importantly, statins promoted formation of the CD14/CD9 protein complex (Figure 4D), and this occurred in dense fractions rather than in light membrane fractions (Figure 4E). These results suggest that statin treatment causes CD14 to be translocated from rafts to non-raft CD9-enriched microdomains, which are less affected by cholesterol depletion. In addition, these findings provide further confirmation that lipid rafts and TEMs are distinct membrane microdomains, as previously proposed [7]. CD81 is another macrophage tetraspanin closely related to CD9 and was reported to be present in CD14-dependent receptor clusters [25,43]. Although CD81, like CD9, was upregulated by statins, its association with CD14 was minimal and did not increase upon statin treatment (Figure 4, D and E). Because the inflammatory phenotype is more pronounced in CD9/CD81 DKO mice than in CD9 single-KO mice [10,12], it is possible that loss of CD81 adds to the inflammatory process, and that the anti-inflammatory effects of statins are mediated partly by CD81-dependent mechanisms. However, our data suggest that the association of CD14 and CD81 is not as robust as that of CD14 and CD9; the latter interaction was maintained in 0.5% Nonidet P-40 and 1% Triton X-100. We speculate that CD9 is a direct partner of CD14, whereas CD81 may associate indirectly with CD14 within a larger tetraspanin complex.

Besides lowering cholesterol, statins also inhibit the synthesis of isoprenoid intermediates including FFP and GGPP. These intermediates serve as lipid attachments and are required for post-translational modification of small GTPases such as Ras, Rho, and Rac; inhibition of the mevalonate pathway by statins prevents these intracellular signaling molecules from translocating from the cytosol to the plasma membrane, and thereby prevents their activation [18]. In particular, inhibition of geranylgeranylation of Rho, which regulate cytoskeletal reorganization and signaling pathways required for activation of NF-κB, may contribute to the anti-inflammatory effects of statins [17]. Our analysis here of the effects of intermediates including mevelonate, and inhibitors including fluvastatin, is consistent with these hypotheses; the mevalonate pathway, especially geranylgeranylation of GTPases, lead to the downregulation of CD9 and CD81 (Figure 7). Because a Rho-kinase inhibitor increased the levels of these tetraspanins, we conclude that Rho-mediated signaling negatively regulates tetraspanin expression, at least partially. Our previous studies showed that CD9 KO macrophages spread to a greater extent than WT macrophages when stimulated with LPS [10], and that CD9/CD81 DKO macrophages are less motile than WT macrophages [12]. Conversely in this study, upregulation of CD9 and CD81 accompanied decreased cell spreading in RAW264.7 cells treated with statins (Figure 3D and Figure S1). Connection of TEMs to integrins and the underlying cytoskeleton has been well established and, via such connections, tetraspanins including CD9 function to regulate cell spreading and motility [9]. Therefore, we hypothesize that macrophage CD9 (and CD81) is a downstream negative regulator of Rho-mediated cytoskeletal dynamics, and that statins reverse cell spreading and NF-κB activation by blocking the Rho-mediated downregulation of CD9.

**Figure 8:**
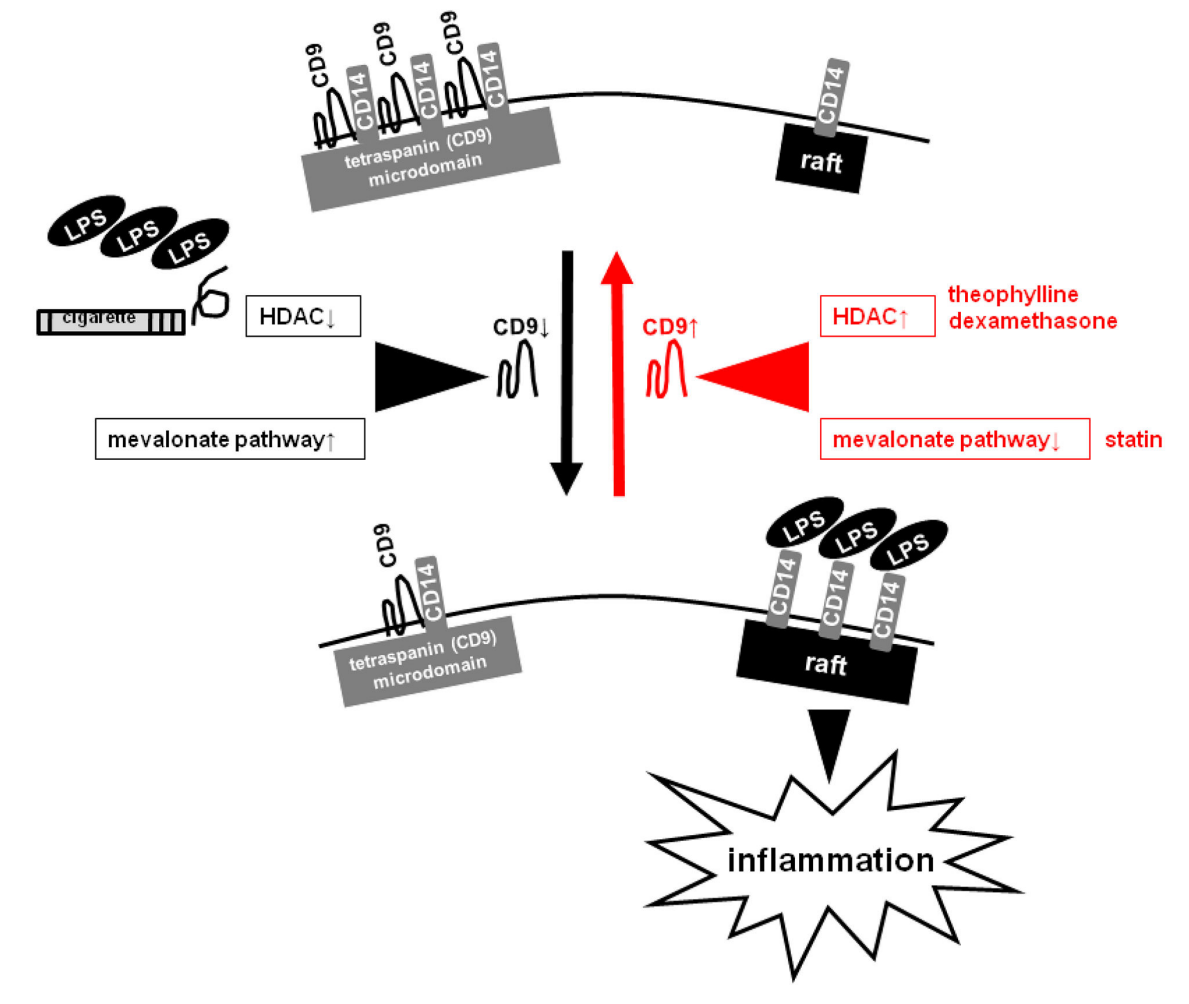
A schematic model illustrating CD9-dependent regulation of LPS-induced inflammatory signaling. CD9 expression is downregulated by either inactivation of HDAC (e.g., by LPS or cigarette smoke exposure) or activation of the mevalonate pathway. The loss of CD9 causes the transfer of the LPS-signaling mediator CD14 from tetraspanin-enriched microdomains to lipid rafts and thereby leads to augmentation of LPS-induced inflammatory signaling in macrophages. Theophylline and dexamethasone (which activate HDACs) or statins (which inhibit the mevalonate pathway) upregulate CD9 and may reverse this cascade in inflammatory lung diseases, including COPD.

The results described here raise two intriguing possibilities. First, functional deficiency of the tetraspanin CD9 might be a common mechanistic component underlying inflammation caused by inactivation of HDAC (e.g., LPS or cigarette smoke exposure) and inflammation induced by abnormal lipid metabolism (e.g., obesity or metabolic syndrome). Downregulation of CD9 may lead to activation of macrophages and their accumulation in tissues, in pro-inflammatory situations. Second, statins may be useful for treatment of inflammatory lung diseases, including COPD [36]. Clinical studies have reported that statin use is associated with beneficial effects for COPD patients [44], but the evidence is still preliminary. We showed that low concentrations of theophylline and dexamethasone, which are widely used to treat COPD patients, increase CD9 levels in RAW264.7 cells by activation of HDACs [12]. Statins upregulate CD9 by a different mechanism (i.e., inhibition of the mevalonate pathway), and these agents could cooperate to prevent CD14 translocation from CD9 microdomains to lipid rafts, thereby reducing the inflammatory response (Figure 8).

In conclusion, based on previous studies suggesting that reduction in the levels of tetraspanin CD9/CD81 may be an underlying mechanism of COPD progression, we screened a drug library for agents that upregulate macrophage CD9/CD81, and found that statins decrease LPS-induced inflammation in mice by blockade of mevalonate pathway-dependent downregulation of CD9. The results of this study underscore the importance of the negative regulator CD9 in lung inflammation and suggest a novel anti-inflammatory mechanism of statins, thereby providing evidence for the hypothesis that statins are useful to treat COPD and its comorbidities.

## Supporting Information

Table S1 **Screen-positive drugs that upregulate either CD9 or CD81 more than 1.5-fold compared to vehicle in RAW264.7 cells.**
(PDF)

Figure S1 **Fluvastatin and simvastatin prevent cell spreading of RAW264.7.** RAW264.7 cells were untreated (Cont, control) or cultured in the presence of 5 µM fluvastatin (Fluv) or simvastatin (Simv), and then stained and photographed (*upper panel*). Scale bar, 100 µm. Percentages of spread cells were determined according to their longest diameters (*lower panel*). Each bar represents the mean ± SEM. ^⋆⋆^ *P* < 0.01.
(TIF)

Figure S2 **Statins transfer CD14 from lipid rafts into CD9-enriched microdomains.** (**A**) RAW264.7 cells were cultured without LPS stimulation and, after the indicated times, the cells were lysed, and protein levels were examined by immunoblotting. Anti-actin blots show that comparable amounts of protein were loaded in each lane. (**B**) RAW264.7 cells were untreated (C, control) or cultured for 24 h in the absence (L) or presence of 3 µM fluvastatin (FL) and stimulated for 24 h with 0.1 µg/ml LPS. Cell lysates were fractionated by sucrose density gradients, and protein distributions in the fractions were visualized by immunoblotting. Data shown are from one representative of three similar experiments. LM, light membrane fractions; D, dense fractions. (**C**) RAW264.7 cells were cultured in the absence or presence of indicated concentrations of fluvastatin (Fluv), and unstimulated (-) or stimulated for 24 h with 1 µg/ml LPS (+). Integrin β2 subunit, CD9, and CD14 proteins in immunoprecipitates (IP) with anti-CD14 mAb were immunoblotted (IB).
(TIF)

Figure S3 **Activation of HDACs or blockade of the mevalonate pathway increases CD9/CD81 levels.** (**A**) RAW264.7 cells were cultured for 48 h in the absence or presence of indicated concentrations TSA and theophylline (*left*) or dexamethasone (*right*). The cells were lysed, and levels of CD9 were examined by immunoblotting. Anti-actin blots show that comparable amounts of protein were loaded in each lane. (**B**) RAW264.7 cells were untreated (-) or cultured for 24 h in the presence of fluvastatin (Fluv) or simvastatin (Simv) (+) in the absence (V, vehicle) or presence of farnesyl pyrophosphate (FPP) or mevalonate. Levels of CD9 and CD81 were examined by immunoblotting. (**C**) RAW264.7 cells were untreated (-) or treated with fluvastatin or simvastatin (+) in the absence (V) or presence of FPP or mevalonate and stimulated for 15 min with 0.1 µg/ml LPS (+). The cells were lysed, and levels of IκBα were examined by immunoblotting.
(TIF)
