# Supplementary material for: Statins Decrease Lung Inflammation in Mice by Upregulating Tetraspanin CD9 in Macrophages
Source: PLoS One. 2013 Sep 9;8(9):e73706. doi: 10.1371/journal.pone.0073706 (PMC3767596; doi:10.1371/journal.pone.0073706)
Supplement: Table S1 — Screen-positive drugs that upregulate either CD9 or CD81 more than 1.5-fold compared to vehicle in RAW264.7 cells. (PDF) [file pone.0073706.s001.pdf]

Table S1. Screen-positive drugs that upregulate either CD9 or CD81 more than 1.5-fold compared to vehicle in RAW264.7 cells.

	CD9	CD81
non-steroidal anti-inflammatory agent		
flufenamic acid	1.56	0.96
anesthetic		
tetacaine hydrochloride	0.93	2.51
antidepressant		
fluvoxamine	1.26	1.64
amitriptyline hydrochloride	1.43	1.83
adrenergic vasoconstrictor		
tetrahydrozoline hydrochloride	0.99	1.82
antihistamine agent		
chlorcyclizine hydrochloride	1.22	1.68
antiarrhythmic agent		
quinidine gluconate	0.94	1.74
quinidine sulfate	1.12	1.60
diuretic		
triamterene	1.52	1.48
statin		
fluvastatin	1.99	1.94
simvastatin	1.42	1.74
mucolytic agent		
acetylcysteine	1.37	1.81
antigout agent		
colchicine	2.22	2.69
sulfonylurea		
glibenclamide	1.32	1.63
chlorpropamide	1.51	1.48
immunosuppressant		
mycophenolic acid	2.11	3.55
estrogen receptor modulator		
raloxifene hydrochloride	1.19	1.53
antitumor agent		
methotrexate	1.87	4.06
dactinomycin	2.13	3.57
mitomycin C	1.33	2.25
cytarabine	2.78	2.54

daunorubicin	2.44	1.94
fluorouracil	1.55	4.78
vinblastine sulfate	4.53	2.94
etoposide	0.82	2.44
floxuridine	1.61	2.63
mitoxanthrone hydrochloride	1.83	1.30
doxorubicin	1.29	1.91
epirubicin hydrochloride	1.30	2.35
antibiotic		
amoxicillin	2.21	1.85
chloramphenicol	1.26	1.93
antifungal agent		
ciclopirox olamine	2.86	2.21
antiparasitic agent		
pyrimethamine	3.04	8.49
pyrvinium pamoate	3.74	4.42
mefloquine	1.41	1.83
niclosamide	2.24	0.85
antiviral agent		
trifluridine	1.00	1.85
antiseptic		
sanguinarine sulfate	1.02	1.53
benzethonium chloride	1.06	1.70
bithionate sodium	0.89	2.00
gentian violet	1.66	2.97
hexachlorophene	1.51	2.16
methylbenzethonium chloride	3.96	2.31
acriflavinium hydrochloride	1.31	1.51
